# Anesthetic Management of Emergent Cesarean Delivery in a Patient With Severe Rheumatic Mitral Stenosis and Cardiopulmonary Complications: A Case Report

**DOI:** 10.7759/cureus.114893

**Published:** 2026-08-20

**Authors:** Arjun Haresh Ghodasara, Mariana Acosta, Tiago de Almeida Macruz, Ally Citor, Mathias Bruefach III

**Affiliations:** 1 Department of Medicine, University of Miami, Miami, USA; 2 Department of Anesthesiology, University of Miami, Miami, USA

**Keywords:** cesarean delivery, dural puncture epidural, extracorporeal membrane oxygenation, mwho class iv cardiac disease, obstetric anesthesia, pulmonary hypertension, severe mitral stenosis

## Abstract

Critical rheumatic mitral stenosis complicated by pulmonary hypertension and right ventricular dysfunction during pregnancy represents one of the most challenging scenarios in obstetric anesthesia. These patients are classified as modified World Health Organization (mWHO) Class IV, which is the highest maternal risk category with high maternal mortality rates. The peripartum period is especially dangerous since even modest hemodynamic shifts can trigger cardiovascular collapse.

We report a successful case of anesthetic management of a 40-year-old gravida 3, para 2 at 29 weeks and three days gestation with severe rheumatic mitral stenosis, moderate-to-severe pulmonary hypertension, moderate-to-severe tricuspid regurgitation, and severely reduced right ventricular function. She was classified as New York Heart Association (NYHA) Class III, mWHO Class IV, and American College of Cardiology/American Heart Association (ACC/AHA) Stage C heart failure. She presented with worsening dyspnea and fetal decelerations after admission for decompensated heart failure and then underwent emergent cesarean section delivery, classified as category 2, with bilateral salpingectomy for permanent sterilization at the patient’s request.

The anesthetic plan centered on three major strategies: a dural puncture epidural (DPE) with incremental dosing to allow gradual sympathectomy, invasive hemodynamic monitoring with early vasopressor support to preserve systemic vascular resistance (SVR), and prophylactic femoral arterial and venous sheath placement to allow rapid escalation to venoarterial (VA) extracorporeal membrane oxygenation (ECMO) if needed. Fortunately, the patient was hemodynamically stable throughout the procedure, did not require mechanical circulatory support, and was discharged in stable condition on postoperative day 10.

This case demonstrates that favorable maternal outcomes can be achieved in patients with mWHO Class IV cardiac disease with meticulous multidisciplinary planning and an anesthetic strategy that prioritizes hemodynamics.

## Introduction

Cardiovascular disease accounts for approximately 27-33% of pregnancy-related deaths in the United States [1]. Severe mitral stenosis with pulmonary hypertension is among the most challenging conditions to manage in pregnancy and is classified as modified World Health Organization (mWHO) Class IV, which is the highest maternal risk category during pregnancy [2]. Maternal mortality in this population can reach 9%, with severe maternal complications occurring in more than 20% of cases [3].

Rheumatic heart disease (RHD) is a leading cause of mitral stenosis in pregnant women and the most common valvular lesion in women of childbearing age [4]. The physiologic changes associated with pregnancy, such as increased blood volume, elevated cardiac output, and a higher resting heart rate, are poorly tolerated in severe mitral stenosis [4]. The fixed mitral orifice cannot accommodate the transvalvular flow, leading to elevated left atrial pressure, pulmonary hypertension, and progressive right heart dysfunction. Delivery and the immediate postpartum period are particularly high-risk because autotransfusion from uterine contractions, relief of aortocaval compression, and rapid fluid shifts can trigger acute pulmonary edema or cardiovascular collapse [4].

Anesthetic management in these patients requires close attention to hemodynamic goals, advanced monitoring, and contingency planning for rapid decompensation. The American College of Cardiology (ACC) and the American Heart Association (AHA) recommend that pregnant women with severe valve disease be monitored at a tertiary care center by a multidisciplinary team of cardiologists, cardiac surgeons, anesthesiologists, and maternal-fetal medicine obstetricians experienced in managing high-risk cardiac pregnancies [5].

We report successful anesthetic management of an emergent cesarean delivery in a patient with severe rheumatic mitral stenosis, pulmonary hypertension, and right ventricular dysfunction, with emphasis on the use of a dural puncture epidural (DPE) technique and standby extracorporeal membrane oxygenation (ECMO) with prophylactic femoral vascular access after careful discussion with the multidisciplinary team. Written informed consent for the publication of this case report was obtained.

## Case presentation

We present a 40-year-old, BMI 25.27 kg/m², G3 P2002 woman at 24 weeks and five days gestation who was admitted to the obstetric emergency department with progressive dyspnea and lower extremity edema. On admission, her blood pressure was 118/81 mmHg, heart rate was 113 beats/min, respiratory rate was 38 breaths/min, oxygen saturation was 100%, and body temperature was 36.5 °C (Table 1). She had a history of severe mitral stenosis of rheumatic etiology, which was initially diagnosed three years earlier. Mitral valve repair was considered, but the patient lost follow-up and received no cardiac management until this admission. At presentation, she exhibited signs of worsening volume overload and declining heart function, consistent with decompensated valvular heart disease. At the time of admission, she was only taking prenatal vitamins.

**Table 1:** Vital signs recorded upon admission to the emergency department and the cardiac care unit

Vitals	Emergency admission	Cardiac care unit admission
Blood Pressure (mmHg)	118/81	98/70
Heart Rate (beats per minute)	113	107
Respiratory Rate (breaths per minute)	38	22
SpO_2_ (%)	100	99
Temperature (°C)	36.5	36.5

Regarding her past medical history, three years before the current admission, the patient sought evaluation by a cardiologist after complaints of worsening shortness of breath and lower extremity edema. Transthoracic echocardiography (TTE) and transesophageal echocardiography (TEE) revealed severe left atrium dilation with a small, layered thrombus, mild right atrial dilation, a rheumatic-appearing mitral valve with severe mitral stenosis, and severe tricuspid regurgitation. Surgical intervention was recommended, but the patient was subsequently lost to follow-up. She reported that she did not return because she was afraid.

At the current admission, the patient reported progressive dyspnea for approximately one year, with her symptoms getting worse in the preceding week. She denied chest pain or palpitations. Her obstetric history included two prior uncomplicated term spontaneous vaginal deliveries, occurring 17 years and 10 years before the current pregnancy. However, she reported experiencing shortness of breath during her second pregnancy. Further details about the patient's previous pregnancies could not be obtained from the available clinical records.

**Figure 1:**
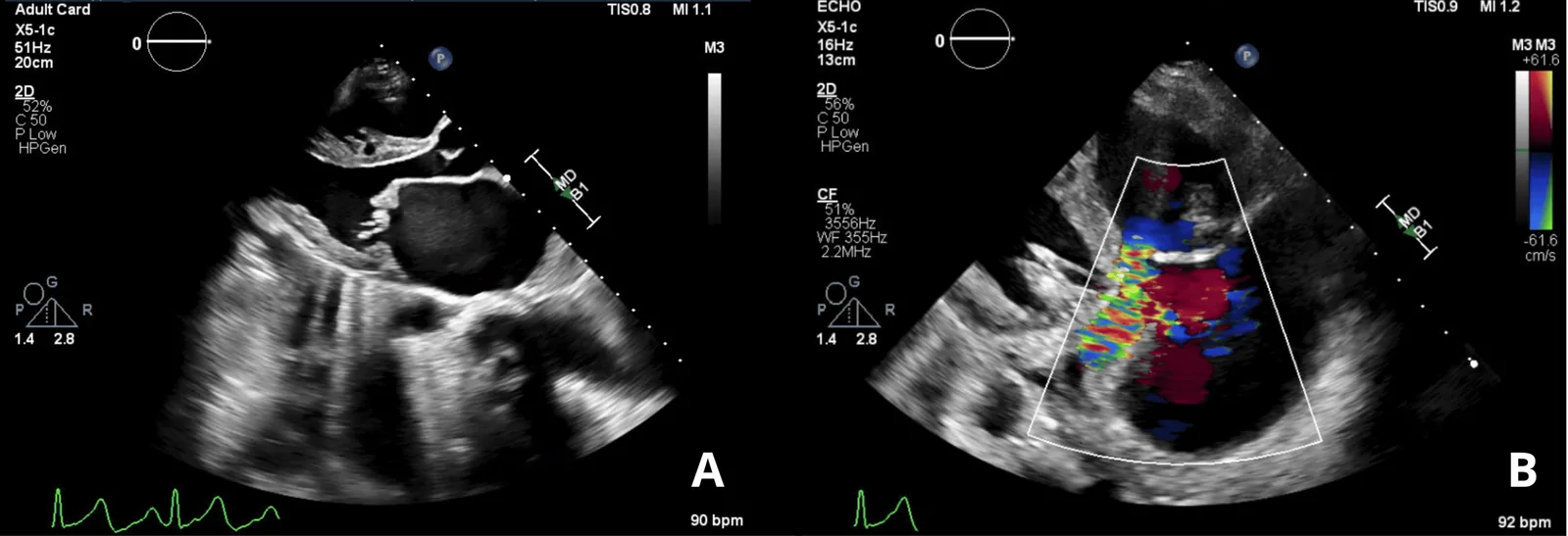
Transthoracic echocardiography in the parasternal long-axis view demonstrating mitral stenosis A: two-dimensional echocardiographic image demonstrating thickening and calcification of the mitral valve leaflets with restricted opening, resulting in the characteristic "hockey stick" deformity; B: color Doppler flow mapping demonstrating accelerated turbulent transmitral flow with aliasing across the mitral valve into the left ventricle, consistent with a markedly elevated transmitral gradient due to significant valvular obstruction.

A TTE (Figure 1) performed on admission demonstrated severe rheumatic mitral valve stenosis with mild to moderate regurgitation, along with moderate to severe tricuspid regurgitation, and an estimated right ventricular systolic pressure (RVSP) of approximately 70 mmHg. The right ventricle was significantly dilated and had reduced systolic function, with a left ventricular ejection fraction (LVEF) of 50-55%. The left atrium was also enlarged, showing layering echogenicity indicative of a thrombus, which prompted initiation of therapeutic anticoagulation with enoxaparin. Bilateral pleural effusions and a small pericardial effusion were also identified.

The patient was classified as New York Heart Association (NYHA) Class III [6], mWHO Class IV, and ACC/AHA Stage C heart failure. She was not considered a candidate for percutaneous mitral balloon valvuloplasty because of her Wilkins score of 13 [7], moderate mitral regurgitation, and the presence of a left atrial thrombus.

She was admitted to the cardiac care unit (CCU) for management of decompensated heart failure (Table 1). Given her extremely high maternal cardiovascular risk, a multidisciplinary team determined that delivery should be planned at 34 weeks of gestation. The team's initial planning was a cesarean section with bilateral tubal ligation, as vaginal delivery was contraindicated due to severe right ventricular dysfunction and pulmonary hypertension. Neuraxial anesthesia was preferred if her condition stayed stable, but general anesthesia would be used if she became unstable or if recent anticoagulation made neuraxial anesthesia unsafe. An arterial line and central venous catheter were planned for close hemodynamic monitoring. Given her high risk for cardiovascular collapse, the cardiac surgery and ECMO teams were on standby with prophylactic femoral access in place for emergency cannulation if needed. Medical management until delivery included metoprolol for heart rate control, furosemide for diuresis, magnesium supplements, and therapeutic enoxaparin for the left atrial thrombus. Furthermore, it was planned, due to the patient's condition, that the teams would also be prepared for postpartum care with a cardiac operating room if unstable or the labor and delivery room if stable. Postoperative admission was planned in either the CCU or cardiac intensive care unit (CICU) depending on the patient's hemodynamic status. 

A follow-up echocardiogram, performed 32 days after admission, demonstrated progression to critical mitral stenosis, with a mitral valve area (MVA) of 0.38 cm² by the pressure half-time method and 0.99 cm² by planimetry, as well as a mean transvalvular gradient of 38.3 mmHg. Severe right ventricular dysfunction persisted. On the same day, right heart catheterization confirmed moderate-to-severe pulmonary hypertension, with a mean pulmonary artery pressure (PAP) of 40 mmHg, a pulmonary capillary wedge pressure (PCWP) ranging from 20 to 28 mmHg, and a pulmonary vascular resistance (PVR) of 9.9 wood units. These hemodynamic findings were consistent with combined pre- and post-capillary pulmonary hypertension. Chest radiography was performed with abdominal shielding and demonstrated pulmonary edema and a moderate right-sided pleural effusion.

Despite the original plan to proceed with delivery at 34 weeks of gestation, the patient's clinical deterioration precluded this timeline. On the 33rd day following her initial hospital admission, at 29 weeks and three days of gestation, she developed worsening dyspnea, regular uterine contractions, and a prolonged fetal heart rate deceleration detected during non-stress testing. Given the progressive maternal clinical deterioration and concerns regarding fetal well-being, the multidisciplinary team decided to proceed with an emergent cesarean delivery in a controlled setting with full multidisciplinary support. The cesarean delivery was classified as a category 2 emergency.

The cardiac anesthesia team evaluated the patient and classified her as American Society of Anesthesiologists (ASA) Physical Status IV. Hemodynamic management was tailored to the patient’s severe mitral stenosis, pulmonary hypertension, and right ventricular dysfunction. Heart rate was targeted between 60 and 80 beats per minute (BPM) to optimize diastolic filling across the stenotic mitral valve. Systemic vascular resistance (SVR) was maintained at the patient’s baseline to preserve coronary blood flow, particularly to the right ventricle. Myocardial depression was to be avoided and also given to prevent factors known to exacerbate pulmonary hypertension, such as hypoxia, hypercarbia, acidosis, pain, and hypothermia.

Preload management was carefully balanced to avoid hypovolemia and volume overload. Given the high risk of pulmonary edema, a restrictive fluid strategy was employed. No intravenous fluid bolus was administered before neuraxial blockade. Crystalloids were only used as medication carriers and replacements for insensible fluid loss. Blood pressure was primarily supported by vasopressors rather than volume expansion. 

In addition to standard ASA monitors, invasive hemodynamic monitoring was established with a left radial arterial line for continuous blood pressure and cardiac output monitoring. Central venous access was obtained through the right internal jugular vein, and two large-bore peripheral intravenous catheters were also secured. Small sheaths were also placed in the left femoral vein and artery as placeholders for emergent venoarterial (VA) ECMO cannulation if required. The cardiac surgery, ECMO, and perfusion teams remained on standby throughout the procedure, and transesophageal echocardiograms were available if needed.

From an anticoagulation standpoint, neuraxial anesthesia was considered acceptable after confirming that an appropriate interval had elapsed since the patient’s last dose of therapeutic enoxaparin. The team chose a carefully titrated neuraxial technique rather than general anesthesia.

With the patient seated upright and after sterile preparation, a DPE was performed at the L3-L4 interspace using loss of resistance to saline. The dura was intentionally punctured with a 27-gauge pencil-point spinal needle to confirm the epidural space. However, no intrathecal medication was given. An epidural catheter was then threaded and secured.

After negative aspiration was confirmed, 2% lidocaine with epinephrine 1:200,000 was administered through the epidural catheter in 3 to 5 mL increments over 15 to 20 minutes. Blood pressure, heart rate, and sensory level were monitored throughout. Norepinephrine was started at 0.05 µg/kg/min and titrated to maintain mean arterial pressure within 10% of the baseline and heart rate between 60 and 80 BPM.

Once a T4-T6 sensory level was achieved, the patient was positioned supine with left uterine displacement. Fetal heart tones were confirmed, and a Foley catheter was placed. The patient received 2 grams of intravenous cefazolin for surgical prophylaxis and 4 grams of intravenous magnesium sulfate for fetal neuroprotection.

A low transverse cesarean section was performed, and a live infant was delivered. The umbilical cord was clamped and cut, and the infant was given to the neonatal team for evaluation and admission to the neonatal intensive care unit (NICU). The infant had a birth weight of 1,370 g and Apgar scores of 4, 8, and 8 at one, five, and 10 minutes (Table 2). Following birth, the infant remained in the NICU for approximately 49 days.

**Table 2:** Umbilical cord blood gas analysis results PCO₂: partial pressure of carbon dioxide; pO₂: partial pressure of oxygen; Hgb: hemoglobin

Variables	Reference value	Results
pH arterial	7.26 – 7.28 ± 0.05	7.20
pH venous	7.35 ± 0.05	7.21
PCO_2 _arterial	49.00 - 52.00 ± 8.00 mmHg	53.00 mmHg
PCO_2 _venous	38.00 ± 6.00 mmHg	58.00 mmHg
PO_2 _arterial	17.00 – 18.00 ± 6.00 mmHg	10.00 mmHg
PO_2 _venous	29.00 ± 6.00 mmHg	13.00 mmHg
Base excess arterial	- 3.00 to + 3.00 mmol/L	- 8.00 mmol/L
Base excess venous	- 3.00 to + 3.00 mmol/L	- 5.00 mmol/L
Hgb O_2_ saturation arterial	20 to 25%	18%
Hgb O_2_ saturation venous	50 to 65%	21%

Uterotonic selection was carefully considered given the patient’s severe pulmonary hypertension and right ventricular dysfunction. Oxytocin was administered after the umbilical cord was clamped and before the placenta was delivered, as an initial 1 IU (international unit) intravenous bolus, followed by an infusion at approximately 10 IU/h, for a total dose of approximately 13 IU. Oxytocin was prepared as 30 IU in 500 mL of intravenous solution, and the total administered dose was approximately 13 IU. Bilateral salpingectomy was subsequently performed for permanent contraception at the patient’s request. 

The patient remained hemodynamically stable throughout the procedure. Heart rate ranged from 80 to 100 beats per minute in sinus rhythm, blood pressure ranged from 85-115/50-75 mmHg on low-dose norepinephrine, and oxygen saturation was maintained at 98-100% on supplemental nasal cannula. There were no acute signs of right ventricular failure or pulmonary edema. The gradual titration of epidural anesthesia helped avoid abrupt hemodynamic shifts, and ECMO was ultimately not required.

The total intraoperative fluid administration was 1,000 mL, estimated blood loss was 500 mL, and urine output was 400 mL. No blood transfusion was required.

The patient was subsequently transferred to the cardiovascular intensive care unit for close postoperative observation. She required short-term vasopressor support with vasopressin and norepinephrine, which was gradually weaned over 24 to 48 hours. She also developed leukocytosis postoperatively and was started empirically on cefepime for concern of hospital-acquired pneumonia. However, bacterial cultures were performed and were negative. On postoperative day three, the patient underwent right-sided thoracentesis that drained 880 mL of transudative pleural fluid.

**Figure 2:**
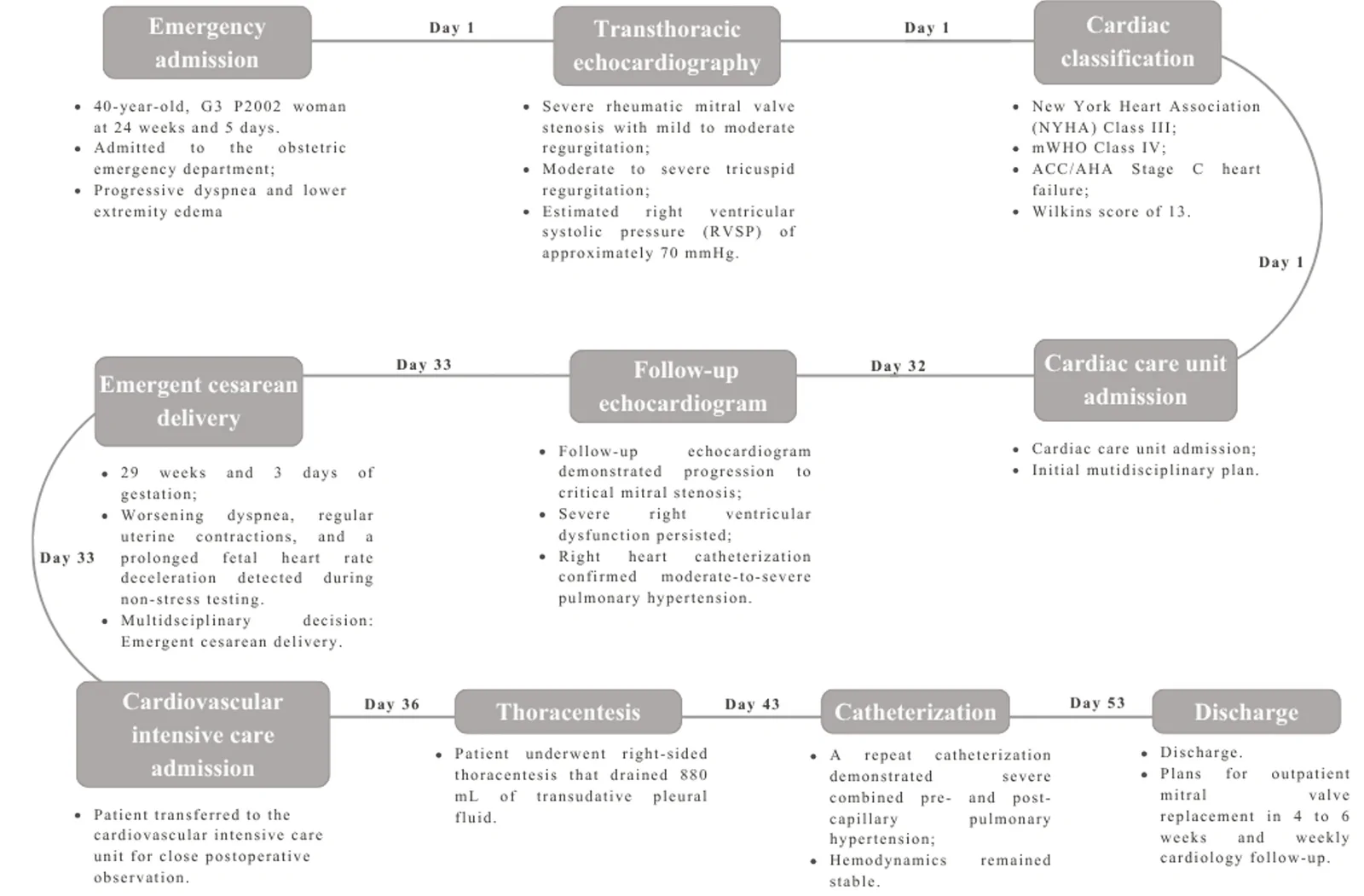
Timeline of the patient’s clinical course, including major diagnostic findings, multidisciplinary management, interventions, and outcomes from emergency admission to hospital discharge The figure was created using Canva (Canva Pty Ltd, Surry Hills, NSW, Australia).

A repeat right heart catheterization 10 days postpartum demonstrated severe combined pre- and post-capillary pulmonary hypertension, but her hemodynamics remained stable. Her medication regimen was adjusted to magnesium oxide 800 mg three times daily, metoprolol 12.5 mg twice daily, digoxin 250 mcg daily, ivabradine 5 mg twice daily, and furosemide 40 mg alternating between once and twice daily. She was discharged on postcatheterization day 10 in stable condition, with plans for outpatient mitral valve replacement in four to six weeks and weekly cardiology follow-up (Figure 2).

## Discussion

This case illustrates the challenges of providing anesthetic care during cesarean delivery in patients with mWHO Class IV cardiovascular disease, and the favorable outcome reflects careful adherence to several key management principles.

Our patient met multiple criteria for mWHO Class IV, including severe mitral stenosis (MVA 0.38-0.99 cm²), moderate-to-severe pulmonary hypertension (PAP 40 mmHg, PCWP 20 mmHg, PVR 9.9 Wood units), NYHA Class III symptoms, and severely reduced right ventricular function. Although any one of these conditions alone would place her in mWHO Class IV, she had all four, placing her at the extreme end of an already high-risk population. The 2022 European Society of Cardiology (ESC) and European Respiratory Society (ERS) Guidelines for Pulmonary Hypertension are generally recommended in women with poorly controlled disease, higher risk profiles, or evidence of right ventricular dysfunction, all of which applied to this patient [8].

The magnitude of maternal risk in this population is well documented. The European Society of Cardiology Registry of Pregnancy and Cardiac Disease (ROPAC) reports a 9% maternal mortality rate among women with pulmonary arterial hypertension, the highest of any cardiac condition studied [8]. Likewise, a recent single-center retrospective cohort study of 32 mWHO Class IV pregnancies echoed this trend, with 65.6% of women experiencing cardiac complications during pregnancy, 75% delivering preterm, and 87% of neonates requiring NICU admission [9]. Overall, more than 20% of these women suffer serious complications, and their risk of death is roughly 18 times higher than that of pregnant women without underlying heart disease [10].

Despite these sobering outcomes, early recognition and coordinated multidisciplinary care can substantially improve maternal and fetal prognosis [2]. In the present case, the patient received coordinated care from maternal-fetal medicine, advanced heart failure, cardiac surgery, cardiac anesthesia, and critical care, and they jointly developed contingency plans for both scheduled and emergent delivery while ensuring immediate access to advanced therapies, including ECMO, if clinical deterioration occurred.

Although vaginal delivery is generally preferred for most cardiac patients because it is associated with less blood loss, lower infection rates, and a reduced risk of thromboembolic complications. However, cesarean delivery is recommended in patients with severe pulmonary arterial hypertension and should also be considered in those with severe mitral stenosis [2].

Several pathophysiologic considerations supported cesarean delivery in our patient. First, the Valsalva maneuver during the second stage of labor causes a significant reduction in preload, which patients with severe mitral stenosis are unable to tolerate because the stenotic valve cannot accommodate abrupt changes in transvalvular flow [2]. Immediately after delivery, uterine contraction returns approximately 500 to 800 mL of blood back into the systemic circulation. When combined with the relief of aortocaval compression, the sudden shift in volume can overwhelm a patient with mitral stenosis, raising left atrial and pulmonary pressures and triggering acute pulmonary edema and right heart failure [2].

In addition, the sustained physiologic stress of labor, including elevations in heart rate, blood pressure, and cardiac output, is dangerous in patients with severe mitral stenosis. Tachycardia shortens diastolic filling time, which is the only window during which blood can cross the stenotic valve. This raises transmitral gradients and left atrial pressure, predisposing the patient to pulmonary edema and acute decompensation. The rise in cardiac output during labor, which can be 50-80% above baseline, further elevates transmitral gradients and compounds this risk [11]. In this case, cesarean delivery also allowed for better coordination with the multidisciplinary team, tighter control of anesthetic depth and hemodynamics, and immediate availability of ECMO if decompensation occurred [2].

However, cesarean delivery itself does carry increased morbidity in cardiac patients. In a retrospective cohort study of over 14,000 delivery hospitalizations among patients with cardiomyopathies, actual cesarean delivery was associated with approximately two-fold higher odds of severe maternal morbidity compared to vaginal delivery [12]. Notably, when comparing intended vaginal delivery versus planned cesarean delivery, there was no significant difference in outcomes [12]. This suggests the elevated risk is associated with cesarean delivery itself, particularly when performed emergently [12]. Therefore, the choice between vaginal and cesarean delivery should be individualized based on the patient's underlying cardiac disease, obstetric circumstances, and the resources available at the delivering institution.

When considering anesthetic management, the choice between neuraxial and general anesthesia for cesarean delivery in high-risk cardiac patients should be individualized on a case-by-case basis, but neuraxial anesthesia is generally preferred, even for patients who are mWHO Class III-IV. It offers several advantages such as avoiding airway manipulation and the hemodynamic challenges related to intubation and extubation. Neuraxial anesthesia also allows for maternal participation in delivery and provides superior post-operative analgesia. The main concern is that sympathetic blockade from neuraxial anesthesia can cause hypotension and reflex tachycardia, which can lead to cardiovascular collapse in patients with limited reserve [2]. 

General anesthesia is typically reserved for patients with cardiopulmonary decompensation requiring intubation. It is also indicated when neuraxial anesthesia is contraindicated, such as following recent anticoagulation, in the setting of coagulopathy, or when severe dyspnea prevents the patient from lying flat [2]. 

The choice of neuraxial technique is critically important in patients with severe cardiac disease. Single-shot spinal anesthesia can produce an abrupt sympathectomy, resulting in marked reductions in SVR and mean arterial pressure. In patients with right ventricular dysfunction, it may compromise coronary perfusion, precipitate right ventricular ischemia, and initiate a cascade of worsening ventricular function, reduced cardiac output, and cardiovascular collapse [13].

For this reason, a slowly titrated neuraxial technique is generally preferred in obstetric patients with pulmonary hypertension. Recommended approaches include a carefully titrated epidural, combined spinal epidural (CSE), or DPE, each allowing greater hemodynamic control than single-shot spinal anesthesia [2].

The DPE technique was selected for this patient, which has gained popularity within obstetric anesthesia for high-risk cardiovascular patients. The technique involves puncturing the dura with a spinal needle to confirm the epidural space before threading an epidural catheter, which creates a small conduit that allows epidural medications to cross into the intrathecal space [14]. A randomized trial involving 150 patients compared DPE, standard epidural, and CSE for cesarean delivery [15]. The results showed that DPE led to a faster onset and better block quality than the standard epidural while having less effect on maternal hemodynamics than CSE [15]. Although CSE provides rapid and dense anesthesia, it produces a more pronounced sympathectomy than DPE, with a correspondingly higher risk of hypotension and greater phenylephrine requirements [15]. Overall, DPE provides an attractive middle ground that combines the reliability of dural puncture with the controlled titration of a standard epidural.

Careful intraoperative hemodynamic management was equally essential. One of the primary goals was maintaining a heart rate between 60 and 80 beats per minute. This was important because tachycardia shortens diastolic filling time and increases the transmitral gradient, raising left atrial and pulmonary pressure and predisposing the patient to pulmonary edema and systemic hypotension. The target heart rate was chosen to balance adequate diastolic filling time against the need to maintain cardiac output. The target heart rate was achieved in this patient with metoprolol [16].

Preload management required similar precision. According to the 2020 ACC/AHA Valvular Heart Disease Guidelines, patients with severe mitral stenosis need enough preload to maintain forward flow across the stenotic valve, but even modest excess fluid can quickly trigger pulmonary edema [5]. Consequently, prophylactic fluid loading before neuraxial anesthesia was avoided. In our case, intravenous fluids were administered only in small aliquots when clinically indicated. Blood pressure was maintained primarily through pharmacologic support rather than liberal fluid administration [2].

Maintaining adequate SVR was important to preserve coronary perfusion and right ventricular function. A reduction in SVR decreases right ventricular pressure, potentially initiating a cycle of worsening right ventricular ischemia, impaired contractility, reduced left ventricular preload, and progressive hypotension [13]. To minimize this risk, vasopressor support was started prophylactically during neuraxial block placement rather than when hypotension developed. Current recommendations support titrated infusions of either phenylephrine (0.5 to 0.75 µg/kg/min) or norepinephrine (0.05 to 0.075 µg/kg/min) to maintain mean arterial pressure close to the baseline and heart rate above 60 bpm [2]. Norepinephrine was selected because of its combined alpha-adrenergic vasoconstriction and mild beta-adrenergic inotropy, supporting both coronary perfusion pressure and right ventricular function. In contrast, phenylephrine may induce reflex bradycardia, which was less desirable in this patient given her dependence on an adequate heart rate to maintain cardiac output [13].

Another notable aspect of this case was the use of standby ECMO with prophylactic vascular access. ECMO has become an important rescue therapy for pregnant and postpartum patients with cardiopulmonary failure that is refractory to standard treatment [2]. In a systematic review of maternal cardiac arrest cases, ECMO was associated with 87.7% of successful resuscitations compared to 58.9% without ECMO [17]. Additional studies have reported maternal survival rates ranging from 72% to 79% among obstetric patients requiring venovenous (VV) or VA ECMO for severe cardiopulmonary disease [18,19].

Despite these encouraging outcomes, establishing ECMO support typically requires 60 to 90 minutes, a delay that may be unacceptable in patients at high risk of sudden cardiovascular collapse. To facilitate rapid initiation of VA ECMO if required, prophylactic femoral arterial and venous sheaths were placed before surgery [2,20]. Successful implementation of this strategy also depends on the immediate availability of an experienced multidisciplinary team, including cardiac surgeons, perfusionists, and ECMO specialists. Although formal selection criteria for standby ECMO have not yet been established, recent reports suggest that patients with severe pulmonary hypertension, advanced ventricular dysfunction, and other mWHO Class IV conditions may benefit from this proactive approach [20]. In our patient, the decision to prepare standby ECMO resulted from multidisciplinary discussion and careful consideration of the anticipated benefits, institutional expertise, and the procedure’s inherent risks.

The choice of uterotonic agents also deserves careful consideration in patients with significant cardiovascular disease because these medications may produce important hemodynamic effects. Oxytocin remains the first-line agent for the prevention and treatment of postpartum hemorrhage. However, rapid bolus administration may cause dose-dependent vasodilation, hypotension, and reflex tachycardia. These effects are minimized when it is administered as a slow infusion [2].

Alternative uterotonics were selected according to the patient’s underlying cardiac disease. Carboprost was avoided because it increases PVR and pulmonary arterial pressure, making it contraindicated in patients with pulmonary hypertension due to the risk of acute right ventricular failure and cardiovascular collapse [2,21]. Methylergonovine was also avoided because its potent vasoconstrictive effects may increase systemic and PVR, induce coronary vasospasm, and precipitate pulmonary edema in patients with severe mitral stenosis [22]. Misoprostol, which has a more favorable cardiovascular profile despite being less potent, was available if additional uterotonic therapy became necessary [2]. In this case, uterine tone was successfully maintained with a slow oxytocin infusion, and blood loss remained limited to approximately 500 mL, eliminating the need for additional uterotonic agents.

The immediate postpartum period represents one of the highest-risk phases for women with severe cardiac disease. Relief of aortocaval compression together with autotransfusion from uterine involution produces a sudden increase in venous return that may precipitate pulmonary edema and acute heart failure [2]. Accordingly, current recommendations support prolonged intensive monitoring in patients with high-risk cardiovascular disease. While ICU-level care for at least 72 hours is generally advised, the ESC/ERS Guidelines recommend a minimum of one week of inpatient observation for patients with pulmonary hypertension because maternal mortality remains elevated throughout the early postpartum period [4].

Consistent with these recommendations, our patient was transferred directly to the cardiovascular intensive care unit (CVICU) after cesarean delivery. Although she required short-term vasopressor support, no major cardiovascular complications occurred, and she was discharged on postoperative day 10 in stable condition with plans for elective mitral valve replacement.

Like all case reports, this study has important limitations. As a single-patient experience, its findings cannot be generalized to all pregnancies complicated by mWHO Class IV cardiovascular disease. Maternal outcomes vary considerably according to the underlying cardiac lesion, disease severity, and the expertise and resources available at the treating center [9,10]. Although our patient experienced an excellent outcome, this should not obscure the substantial maternal morbidity and mortality that continue to characterize this population.

In addition, this case was managed at a large tertiary academic center with immediate access to maternal-fetal medicine, advanced heart failure specialists, cardiac anesthesia, cardiac surgery, ECMO, and a dedicated cardiovascular intensive care unit. These resources are not universally available and may have contributed to the favorable outcomes [2,13]. Furthermore, both the use of prophylactic standby ECMO and the selection of DPE were based on limited evidence. Formal guidelines for patient selection for standby ECMO have not yet been established, and comparative studies evaluating neuraxial techniques in patients with severe cardiac disease remain scarce [14,20]. Finally, detailed longitudinal maternal follow-up, comprehensive neonatal outcomes, and continuous intraoperative hemodynamic data were unavailable, and publication bias inherent to case reports should also be acknowledged when interpreting these findings.

## Conclusions

Caring for pregnant patients with mWHO Class IV cardiac disease remains one of the most challenging scenarios in obstetric anesthesia, as morbidity and mortality remain high in this population. This case demonstrates that favorable outcomes may be achieved through individualized anesthetic planning, effective communication, early multidisciplinary coordination of clinical management, and anticipation of potential hemodynamic instability requiring intensive care. Nevertheless, this report has inherent limitations, particularly because it describes the experience of a single patient. Although the case supports the feasibility of a multidisciplinary approach in this complex clinical setting, larger studies are needed to determine whether these findings and management strategies can be generalized to other patients.
